# The Pulse Test

**Published:** 1878-05

**Authors:** 


The PULSE beats about sixty-eight times in every minute of healthful adult life. The range is from sixty-six to seventy-two. When it is below sixty-six, there is something at fault; when it is over seventy-two, during all the hours of the twenty-four, there is always disease; and if it continues so for weeks and months, there is the strongest ground for apprehension that consumption is approaching.

There are intelligent men in the profession who will not coincide with this statement, but it will be because they have not had the opportunities of observation.

Whatever may be said of auscultation, of plessimetry, of sounding, of expectoration, there is in none of these a guide so sure as the condition of the pulse, with the aid of a competent interpreter; more, it is worth, to such an one, all the other modes of determination put together. It is said that the physicians among some of the Orientals are not allowed to see their female patients, the hand only being put out through the bed curtain, and by feeling the pulse, prescriptions must be made. If the powers of life are being pressed to death, the full, soft, slow pulse tells it in an instant; if active, and actual destruction of organic life is taking place in the body, the inflammatory pulse, quick, wiry, angry, spiteful, at once raises the note of alarm. Every physician knows how gratefully the pulsation, as of a woolen yarn beneath his finger, strikes upon his perceptions, on some urgent call, and how troubled if it gives the feeling of a quick vibrating small wire. The multitude of shades of difference between these carry with them their varied impressions, all highly instructive. In strongly-marked cases, however wan the patient may look, however hollow or fierce his cough, at first sight, an instantaneous feeling of the pulse is sufficient for the conclusion, "*You* have no consumption." But inasmuch as there have been cases of no appreciable activity of pulse, and even diminished pulse where consumption existed, the wise physician will never pronounce an opinion on any one single symptom. In some cases of spinal irritation, for example, there may be a troublesome hacking or hemming, and a quick pulse for months and years, without any special disease in the lungs. Still, this one broad fact should stand out prominently as an instructive beacon to all:

*A pulse steadily over eighty beats in a minute, for weeks together, is a forerunner of consumption.*

The physician in his kindness or hopefulness may tell you that some persons have a high pulse constitutionally, hereditarily, or some other plausible reason may be given for its presence in you; but if you are wise, with a pulse among the eighties, you will set it down as consumption begun, and will act accordingly.

